# J-shaped relationship between stress hyperglycemia ratio and 90-day and 180-day mortality in patients with a first diagnosis of acute myocardial infarction: analysis of the MIMIC-IV database

**DOI:** 10.1186/s13098-024-01380-2

**Published:** 2024-06-16

**Authors:** Ben Hu, Xinghua Chen, Yuhui Wang, Xing Wei, Jun Feng, Linlin Hou

**Affiliations:** 1 Department of Cardiology, The Second People’s Hospital of Hefei, Hefei Hospital Affiliated to Anhui Medical University, Hefei, 230011 Anhui China; 2 The Fifth Clinical Medical School of Anhui Medical University, Hefei, 230000 Anhui China; 3 Hefei Hospital Affiliated to Anhui Medical University, Hefei, 230011 Anhui China

**Keywords:** Stress hyperglycemia ratio, Acute myocardial infarction, Mortality, MIMIC-IV database

## Abstract

**Aims:**

The Stress Hyperglycemia Ratio (SHR) potently predicts adverse outcomes in patients with cardiovascular and cerebrovascular diseases. However, the relationship between SHR and short-term mortality risk in patients with a first diagnosis of acute myocardial infarction (AMI) remains contentious. This study sought to understand better the relationship between SHR and short-term mortality risk in patients with a first diagnosis of AMI.

**Methods:**

We conducted a cohort study using data from 1961 patients with a first diagnosis of AMI from the MIMIC-IV (version 2.2) database. Patients were divided into three groups based on SHR tertiles. The Cox proportional hazards model and a two-segmented Cox proportional hazards model were used to elucidate the nonlinear relationship between SHR in patients with a first diagnosis of AMI and mortality.

**Results:**

Of the surveyed population, 175 patients (8.92%) died within 90 days, and 210 patients (10.71%) died within 180 days. After multivariate adjustments, elevated SHR levels were significantly and non-linearly associated with a higher risk of 90-day and 180-day mortality in patients with a first diagnosis of AMI, showing a J-shaped correlation with an inflection point at 0.9. Compared to participants with SHR levels below the inflection point, those with higher SHR levels had a fivefold increased risk of 90-day mortality (hazard ratio [HR] 5.74; 95% confidence interval [CI] 3.19, 10.33) and a fourfold increased risk of 180-day mortality (HR 4.56; 95% CI 2.62, 7.95). In the subgroup analysis, patients with pre-diabetes mellitus (pre-DM) and higher SHR levels had increased 90-day (HR 6.90; 95% CI  1.98, 24.02) and 180-day mortality risks (HR 5.30; 95% CI  1.96, 14.27).

**Conclusion:**

In patients with a first diagnosis of AMI, there is a J-shaped correlation between SHR and 90-day and 180-day mortality, with an adverse prognostic inflection point of SHR at 0.9.

**Supplementary Information:**

The online version contains supplementary material available at 10.1186/s13098-024-01380-2.

## Introduction

As the global population ages and expands, cardiovascular diseases (CVD) have become the leading causes of mortality and morbidity worldwide [1]. However, individuals with cardiovascular diseases often exhibit metabolic disturbances due to unhealthy dietary and lifestyle habits, potentially exacerbating adverse cardiovascular outcomes [2, 3]. Previous research has shown that elevated blood glucose levels upon hospital admission in patients with CVD are independently associated with poor prognosis [4, 5]. However, hyperglycemia often arises from a combination of acute stress and chronic glucose levels and thus might not necessarily reflect an acute rise in glucose. Consequently, some researchers have employed a novel marker of hyperglycemic state, the Stress Hyperglycemia Ratio (SHR), to represent genuine acute hyperglycemia [6].

Stress-induced hyperglycemia is a relative increase in glucose caused by inflammation and neurohormonal disruptions during severe illness. The interaction between hyperglycemia and illness severity partly mirrors the more intense inflammatory and neurohormonal responses seen with more severe disease stimuli [7]. Many recent studies have demonstrated that acute hyperglycemia upon admission in patients with acute coronary syndrome (ACS) is independently associated with early and late adverse prognoses [8]. A study from the COACT registry revealed that SHR is an effective predictor of post-percutaneous coronary intervention (PCI) major adverse cardiac and cerebrovascular events (MACCE), especially in patients with non-diabetic ST-segment elevation myocardial infarction (STEMI) [9]. Yet, another report indicated no significant correlation between SHR bifurcation and short-term survival in diabetic patients [10]. Thus, the relationship between SHR and short-term mortality risk in patients with a first diagnosis of AMI remains contentious and merits further investigation. Moreover, past research has had limitations, including small sample sizes and overlooking potential confounding factors like the influence of other diseases and medication use, which could affect the results. Hence, in this study, using the MIMIC-IV (version 2.2) database, we aim to understand better the relationship between SHR and short-term mortality risk in patients with a first diagnosis of AMI.

## Materials and methods

### Data source and study population

**Fig. 1:**
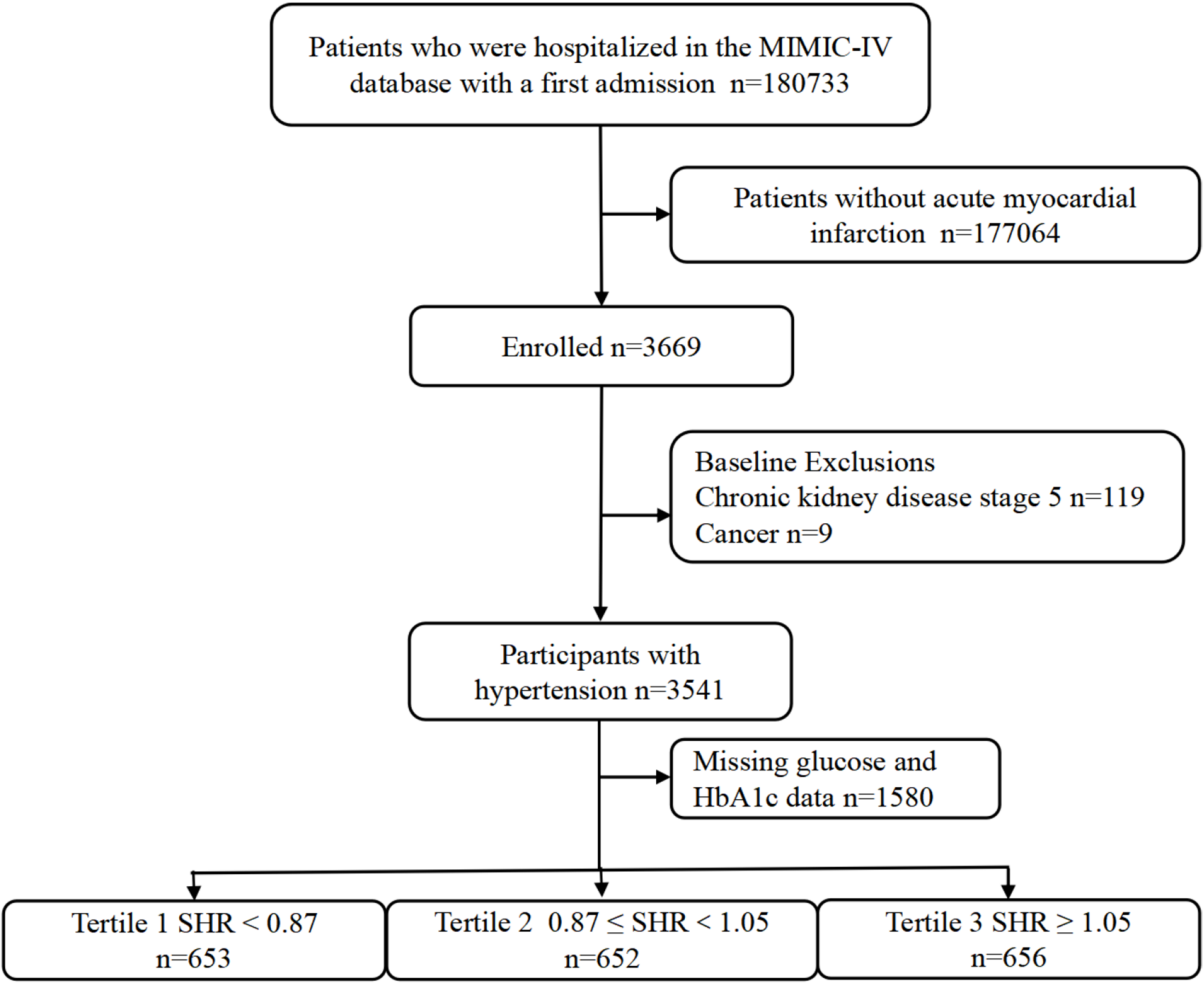
Flow diagram of study

This retrospective study utilized health-related data from the MIMIC-IV (version 2.2) database, a comprehensive large-scale database developed and managed by the MIT Computational Physiology Laboratory. The database comprises over 50,000 high-quality medical records of patients admitted to the Intensive Care Unit (ICU) at the Beth Israel Deaconess Medical Center [11]. Notably, all personal identifying information has been anonymized to safeguard patient privacy. In light of the nature of this study, the Institutional Review Board of the Beth Israel Deaconess Medical Center waived the requirement for informed consent [11]. To access the database, the author (XHC) obtained the necessary certification and then extracted the required variables (Certification No.: 58951192). For this study, patients with a first diagnosis of AMI based on the International Classification of Diseases (ICD), ICD-9, and ICD-10 were included. Participants under the age of 18 years (n = 0), those with chronic kidney disease stage 5 (n = 119), with malignant tumors (n = 9), and those with missing glucose and HbA1c data (n = 1580) were excluded. In the final analysis, 1961 individuals were included and categorized into three groups based on the tertiles of their SHR levels (Fig. 1).

### Data collection

Data extraction was performed using the PostgreSQL tool (v.14, PostgreSQL Global Development Group, Berkley, California, USA). Diagnoses of patients were retrieved as per ICD-9 and ICD-10 using Structured Query Language (SQL). Baseline characteristics, laboratory indicators, and medication usage records were queried through the item IDs stored in MIMIC-IV and matched to selected patients using a unique identifier (subject_id). Extracted variables included: (1) Baseline characteristics: age, sex, height, weight, systemic inflammatory response (SIRS), sequential organ failure assessment (SOFA); (2) Comorbidities: hypertension, cardiogenic shock, atrial fibrillation, cardiac arrest, heart failure, diabetes, chronic kidney disease stage 5, malignant tumor; (3) Laboratory parameters: red blood cell (RBC), white blood cell (WBC), platelet, creatinine, glucose, hemoglobin A1C (HbA1C); (4) Medication use: vasoactive drugs, antihypertensive drugs, antilipidemic drugs, antiplatelets, insulin; (5) Hospital and ICU admission data: admission and discharge dates, ICU admission and discharge dates, death date and time. All measurements utilized in this study were from the initial 24 h post-admission. Specific details and query codes for each metric are available in Table S1.

### Data definitions

The Body Mass Index (BMI) was calculated as weight (kg) divided by the square of height (m). The SHR was determined using the following formula: SHR = admission blood glucose (ABG) (mmol/L)/[1.59 × HbA1c (%) − 2.59] [6]. Diabetes was defined as a history of diabetes or HbA1c > 6.5%. Pre-DM was defined as patients without a history of diabetes but with HbA1c levels between 5.7 and 6.4%. Normoglycemia (NGR) was identified in patients without a history of diabetes or with HbA1c ≤ 5.7% [12].

### Outcomes

The primary outcomes were all-cause mortality at 90 and 180 days post-admission, calculated based on the first AMI diagnosis time and the follow-up death dates from MIMIC-IV 2.2.

### Statistical analysis

Based on the baseline SHR tertiles, data are expressed as mean (SD) or median (interquartile range) for continuous variables and frequency (percentage) for categorical variables. Differences between SHR tertiles groups were assessed using the chi-squared test (for categorical variables), one-way ANOVA (for normally distributed data), and the Kruskal–Wallis H test (for skewed data). The lowest tertile of SHR served as the reference group. To analyze the association between the SHR index and the risk of 90-day and 180-day death, multivariable Cox proportional hazards regression models were utilized to calculate Hazard ratios (HRs) and 95% confidence intervals (CIs). Model 1 did not adjust for any covariates. Any covariate altering the resulting estimate by more than 10% was included in Model 2 as a potential confounder. If the covariate changed the resulting estimate by more than 10% or had a regression coefficient P-value < 0.1, it was considered a potential confounder in Model 3 [13]. To prevent multicollinearity, variables with a variance inflation factor (VIF) greater than five were excluded from the models. The Kaplan–Meier survival analysis was also employed to assess endpoint occurrence rates based on different SHR levels.

Likewise, analyses were divided into groups according to the following factors: age, sex, BMI, hypertension, heart failure, atrial fibrillation, and diabetes. Except for the stratification variable, the adjustment technique was the same as in Model 3. Log-likelihood ratio tests were employed to evaluate interactions between SHR and outcomes between subgroup factors.

Additionally, restricted cubic splines were used with three knots (10th, 50th, and 90th percentiles) to examine dose–response associations. We then calculated HR for SHR and outcomes by running a log-likelihood ratio test for the non-linearity of the smooth curve fit, contrasting the segmented regression model to the single-linear (non-segmented) model while accounting for relevant confounders.

The highest covariate missing was the BMI (29.63%). We employed a multiple imputation and chained equation technique based on five replications in the R MI process to account for the missing data (BMI, WBC, RBC, PLT, and creatinine) in to prevent a deterioration in the effectiveness and bias of the statistical analyses owing to the direct exclusion of missing values [13]. Cox regression analysis was performed on the five newly created data sets, and the outcomes were merged using Rubin's rules [14, 15]. We performed sensitivity analyses to assess how reliable our results were. Firstly, distributions of variables with missing data comparing observed complete case data. Secondly, the association between SHR and the risk of 90-day or 180-day mortality was investigated using data before multiple imputations (n = 1358). Thirdly, further adjustments were made to the SIRS and SOFA scores based on clinical data (n = 986). Finally, recognizing the varying diabetes status among patients, we performed subgroup analyses. All analyses have been conducted using the data after multiple imputations except Table S3, which used raw data for analyses. R 4.3.0 (http://www.R-project.org) was used for all analyses. A two-sided P-value of less than 0.05 was deemed statistically significant.

## Results

### Baseline characteristics of patients with AMI

Data were derived from 1961 AMI patients (mean age 66.65 years; 69.10% male). Those with elevated SHR tended to be older males with increased white blood cell counts and glucose levels. Notable differences in age, sex, white blood cell count, red blood cell count, platelets, creatinine, glucose, HbA1c, heart failure, cardiogenic shock, cardiac arrest, atrial fibrillation, hypertension, diabetes, antilipidemic drugs, antiplatelets, vasoactive drugs, 90-day and 180-day mortality were statistically significant across the three patient groups (all P < 0.05) (Table 1).

**Table 1:** Baseline characteristics of patients according to tertiles of stress hyperglycemia ratio

	Stress hyperglycemia ratio				
Characteristics	Total	Tertile 1 (< 0.87)	Tertile 2 (≥ 0.87, < 1.05)	Tertile 3 (≥ 1.05)	P value
Patients, n	1961	653	652	656	
Age (years)	66.65 (13.22)	67.02 (13.61)	65.88 (12.96)	67.05 (13.06)	< 0.001
Sex %					0.028
Male	1355 (69.10%)	429 (65.70%)	473 (72.55%)	453 (69.05%)	
Female	606 (30.90%)	224 (34.30%)	179 (27.45%)	203 (30.95%)	
BMI kg/m2	29.20 (7.56)	28.89 (8.09)	29.43 (7.09)	29.28 (7.46)	0.234
WBC (1000 cells/uL)	10.85 (4.94)	10.14 (4.80)	10.49 (4.20)	11.91 (5.55)	< 0.001
RBC (1000 cells/uL)	4.30 (0.74)	4.32 (0.72)	4.39 (0.68)	4.19 (0.80)	< 0.001
PLT (1000 cells/uL)	234.38 (78.29)	240.05 (83.66)	240.05 (83.66)	227.65 (75.13)	0.025
Creatinine (mg/dL)	1.26 (0.80)	1.07 (0.50)	1.07 (0.42)	1.22 (0.91)	< 0.001
Glucose (mg/dL)	135.81 (57.54)	111.33 (38.00)	120.04 (30.54)	175.86 (71.37)	< 0.001
HbA1c (%)	6.45 (1.64)	7.07 (2.07)	6.04 (1.10)	6.24 (1.41)	< 0.001
Heart failure %	596 (30.39%)	185 (28.33%)	164 (25.15%)	247 (37.65%)	< 0.001
Cardiogenic shock %	190 (9.69%)	42 (6.43%)	42 (6.44%)	106 (16.16%)	< 0.001
Cardiac arrest %	37 (1.89%)	5 (0.77%)	8 (1.23%)	24 (3.66%)	< 0.001
Atrial fibrillation %	495 (25.24%)	153 (23.43%)	146 (22.39%)	196 (29.88%)	0.003
Hypertension %	919 (46.86%)	299 (45.79%)	331 (50.77%)	289 (44.05%)	0.041
Diabetes %	770 (39.27%)	303 (46.40%)	187 (28.68%)	280 (42.68%)	< 0.001
Antihypertensive drugs %	1904 (97.09%)	633 (96.94%)	631 (96.78%)	640 (97.56%)	0.673
Antilipidemic drugs %	1854 (94.54%)	625 (95.71%)	627 (96.17%)	602 (91.77%)	< 0.001
Antiplatelets %	1891 (96.43%)	635 (97.24%)	633 (97.09%)	623 (94.97%)	0.047
Vasoactive drugs %	701 (35.75%)	213 (32.62%)	208 (31.90%)	280 (42.68%)	< 0.001
Insulin use %	603 (30.75%)	231 (35.38%)	140 (21.47%)	232 (35.37%)	< 0.001
90-day death %	175 (8.92%)	44 (6.74%)	31 (4.75%)	100 (15.24%)	< 0.001
180-day death %	210 (10.71%)	53 (8.12%)	42 (6.44%)	115 (17.53%)	< 0.001

Mean (SD) or median (interquartile range) for continuous variables and as frequencies (percentages) for categorical variables

BMI: body mass index; WBC: white blood cell; RBC: red blood cell; PLT: platelet

### Associations between SHR and outcomes

Over the 90-day and 180-day follow-up periods post-admission, there were 175 and 210 recorded deaths, respectively. After multivariable adjustment for age, sex, BMI, cardiogenic shock, cardiac arrest, and hypertension, compared with the reference tertiles, the third tertiles showed a significant association with SHR concerning 90-day and 180-day mortality in Model 2. With further adjustments for potential confounder, the results were consistent in Model 3, multivariable-adjusted HRs (95% CIs) tertiless of across SHR were 1.00 (reference), 0.81 (0.50, 1.30), and 1.94 (1.33, 2.82) (P trend < 0.001); and 1.00 (reference), 0.88 (0.58, 1.34), and 1.92 (1.37, 2.70) (P trend < 0.001), respectively (Table 2).

**Table 2:** Multivariable Cox regression analyses for 90-day and 180-day mortality in patients with acute myocardial infarction

Outcomes Exposure	Model 1 HR, 95% CI, P	Model 2 HR, 95% CI, P	Model 3 HR, 95% CI, P
90-day mortality			
SHR index	2.99 (2.29, 3.90) < 0.001	2.59 (1.94, 3.48) < 0.001	1.93 (1.42, 2.63) < 0.001
SHR index (tertiles)			
T1	Ref	Ref	Ref
T2	0.70 (0.44, 1.11) 0.127	0.75 (0.47, 1.19) 0.223	0.81 (0.50, 1.30) 0.379
T3	2.40 (1.68, 3.42) < 0.001	2.03 (1.41, 2.92) < 0.001	1.94 (1.33, 2.82) 0.001
P for trend	< 0.001	< 0.001	< 0.001
180-day mortality			
SHR index	2.71 (2.09, 3.50) < 0.001	2.39 (1.80, 3.16) < 0.001	1.85 (1.38, 2.49) < 0.001
SHR index (tertiles)			
T1	Ref	Ref	Ref
T2	0.79 (0.52, 1.18) 0.243	0.85 (0.57, 1.28) 0.450	0.88 (0.58, 1.34) 0.553
T3	2.30 (1.66, 3.19) < 0.001	2.01 (1.44, 2.81) < 0.001	1.92 (1.37, 2.70) < 0.001
P for trend	< 0.001	< 0.001	< 0.001

Model 1: no covariates were adjusted

Model 2: we only adjusted for age, sex (male, female), BMI, cardiogenic shock (yes, no), cardiac arrest (yes, no), hypertension (yes, no)

Model 3: we additionally adjusted for WBC, RBC, PLT, creatinine, diabetes (yes, no), heart failure (yes, no), atrial fibrillation (yes, no), antihypertensive drugs (yes, no), antilipidemic drugs (yes, no), antiplatelets (yes, no), vasoactive (yes, no) and insulin (yes, no)

BMI: body mass index; WBC: white blood cell; RBC: red blood cell; PLT: platelet; SHR: Stress hyperglycemia ratio

**Fig. 2:**
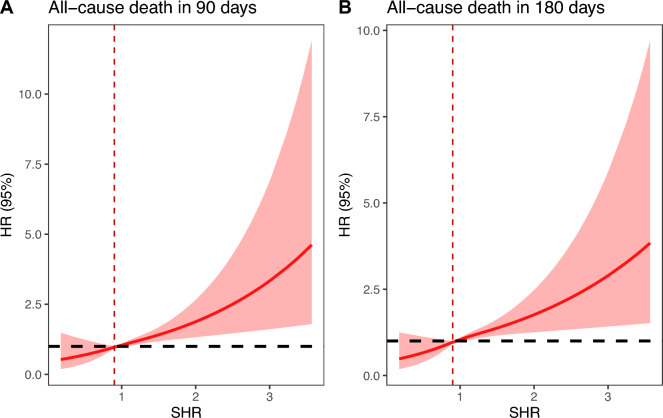
Restricted cubic spline analyses of the association of SHR with all-cause mortality (**A** all-cause death in 90 days, **B** all-cause death in 180 days). Heavy central lines represent the estimated adjusted hazard ratios. The 95% confidence interval is represented by the red band. The adjustment strategy is the same as the Model 3

**Fig. 3:**
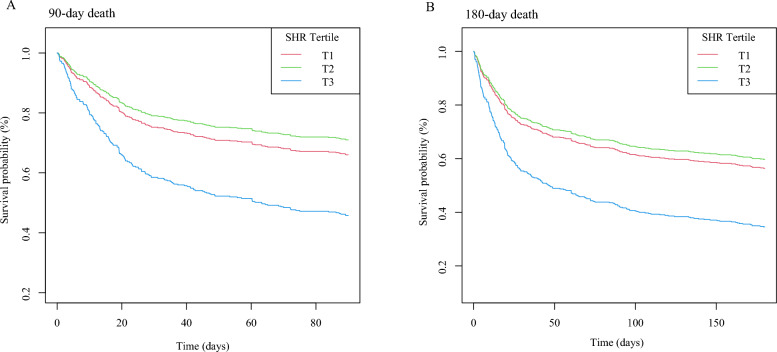
Kaplan–Meier survival analysis curves for all-cause mortality. Footnote SHR index tertiles. Kaplan–Meier curves showing cumulative probability of all-cause mortality according to groups at 90 days (**A**), and 180 days (**B**). The adjustment strategy is the same as the Model 3

The dose–response relationship between SHR and the adjusted hazard ratio for 90-day and 180-day mortality in AMI patients was depicted using restricted cubic splines. A J-shaped association between SHR and the 90-day and 180-day mortality rates was observed (Fig. 2). Additionally, a combination of Cox proportional hazard models with a two-segmented Cox proportional hazards model was employed to study the non-linear relationship between SHR levels in AMI patients and the mortality mentioned above rates (P for log-likelihood ratio < 0.05) (Table 3). An inflection point was detected at an SHR of 0.9. When the SHR exceeds 0.9, for each unit increase in the SHR level, the adjusted HRs for 90-day and 180-day mortality increase by fivefold (HR 5.74; 95% CI, 3.19–10.33) and fourfold (HR 4.56; 95% CI, 2.62–7.95), respectively. However, when the SHR is below 0.9, there is no significant association with 90-day and 180-day mortality. Survival analysis further suggested a positive relationship between baseline SHR levels, when categorized into tertiles, and the 90-day and 180-day mortality rates (Kaplan–Meier, log-rank P < 0.001) (Fig. 3). Similar results were also observed when categorized into two groups according to the inflection point (Figure S1).

**Table 3:** Threshold effect analysis of SHR index on 90-day and 180-day mortality in acute myocardial infarction patients. The adjustment strategy is the same as the Model 3

	Adjusted HR (95% CI), P-value
90-day mortality	
Fitting model by twp-piecewise linear regression	
Inflection point	0.9
SHR index < 0.9	0.37 (0.07, 1.87) 0.229
SHR index > 0.9	5.74 (3.19, 10.33) < 0.001
P for the Log-likelihood ratio	0.009
180-day mortality	
Fitting model by twp-piecewise linear regression	
Inflection point	0.9
SHR index < 0.9	0.56 (0.12, 2.60) 0.463
SHR index > 0.9	4.56 (2.62, 7.95) < 0.001
P for the Log-likelihood ratio	0.033

### Stratified analysis

**Fig. 4:**
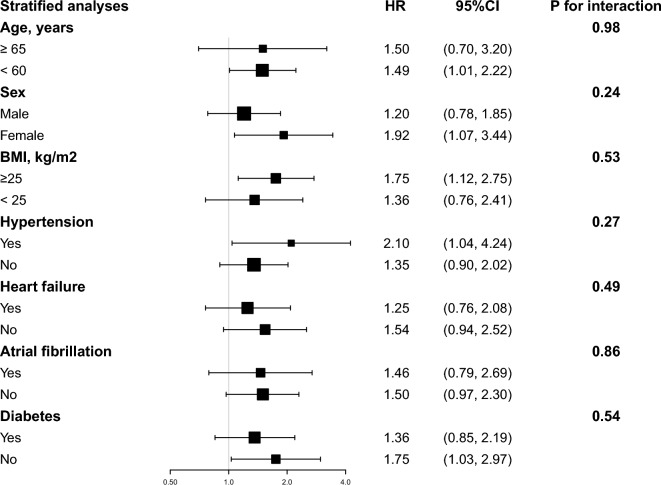
Forest plots of stratified analyses of SHR index and all-cause mortality (90 days). With the exception of the stratified variable itself, the adjustment approach is the same as for Model 3

**Fig. 5:**
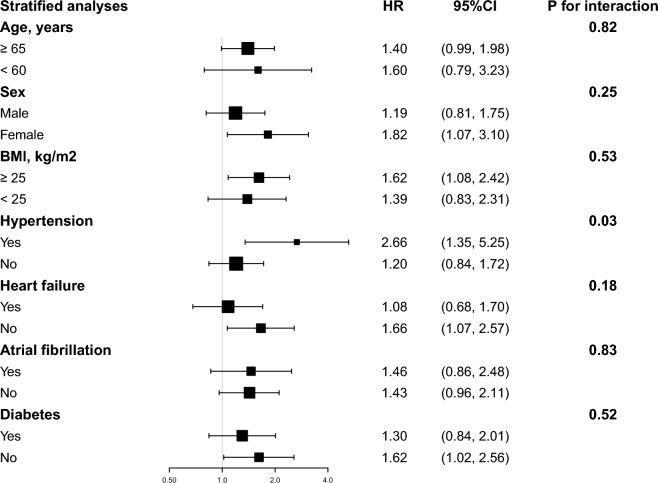
Forest plots of stratified analyses of SHR index and all-cause mortality (180 days). With the exception of the stratified variable itself, the adjustment approach is the same as for Model 3

After stratification of studies by age, sex, BMI, hypertension, heart failure, atrial fibrillation, and diabetes to explore the associations with 90-day and 180-day mortality (Figs. 4 and 5). Consistent results were observed in people under age 60 who were female, BMI (> 25 kg/m^2^), with hypertension, without heart failure. The model's interaction tests for covariates with the SHR were non-significant except for hypertension in 180-day mortality (P for interaction = 0.03) (Fig. 5). Hypertensive patients exhibited a heightened adverse effect from an elevated SHR compared to non-hypertensive individuals.

### Sensitivity analysis

The characteristics of raw data and data after multiple imputations (Table S2). Similar results were observed when we used data before multiple imputations to investigate the association between SHR and the risk of 90-day and 180-day mortality using multivariate Cox regression models (Table S3). Similar results were also observed when further adjusting for SIRS and SOFA (Table S4). When we conducted a subgroup analysis for individuals with different diabetes statuses, the results were consistent among those with pre-diabetes and diabetes (Tables S5 and S6).

## Discussion

Using data from the MIMIC-IV database (version 2.2), our study revealed an independent association between SHR and 90-day and 180-day mortality in patients with a first diagnosis of AMI. This association was especially evident in those with pre-DM. Additionally, we observed a J-shaped curve relationship between SHR levels and 90-day and 180-day mortality for AMI-diagnosed patients. When the SHR was below 0.9, the association was not significant; however, when it exceeded 0.9, a positive correlation emerged. Understanding and optimizing the distribution of SHR levels in AMI patients may facilitate improvements in subsequent health outcomes for these individuals.

Stress hyperglycemia has been identified as a potent predictor of adverse outcomes in ACS patients [16, 17], significantly correlating with adverse cardiovascular and cerebrovascular events [18]. Unlike admission glucose, SHR—when adjusted for background glucose—may be a superior biomarker for severe illness [6]. For instance, a study by Marenzi et al., which included 1553 AMI patients, with the primary endpoint being a composite of cardiogenic shock, acute pulmonary edema, and in-hospital mortality, identified SHR as a better biomarker for in-hospital mortality and morbidity than absolute glucose levels upon admission [19]. Their findings underscored the predictive value of stress hyperglycemia and its role as an independent predictor of post-AMI in-hospital mortality, which can potentially help identify AMI patients at elevated risk for subsequent adverse outcomes. In a longer-term prognosis, Kojima et al. recruited 6287 STEMI patients discharged safely. With endpoints being all-cause mortality and readmission due to heart failure (median follow-up of 1522 days), they found that in non-diabetic patients, those in the highest quartile of SHR faced significantly worse long-term outcomes than those in the lower quartile [20]. In our study, RCS demonstrated a J-shaped relationship between SHR and short-term mortality in AMI patients. Another study involving 5562 ACS patients who underwent PCI reported a U-shaped or J-shaped association between SHR and early and late adverse outcomes [21]. Furthermore, a large cohort study from China demonstrated a J-shaped correlation between SHR and adverse outcomes in diabetes [22].

Our findings align with these previous studies to some extent. Given the above positive relationship between SHR and acute cardiac events, our study's observation of a J-shaped association between SHR and short-term mortality is not unexpected, with an SHR of 0.90 as the inflection point. Moreover, our findings emphasize a strong association between SHR and short-term mortality, especially among pre-DM patients. Compared to diabetic patients, those without diabetes exhibited a significantly heightened in-hospital mortality risk. Such differences have been reported in prior studies. For example, Kerby et al. proposed that stress hyperglycemia correlates closely with mortality in patients without diabetes rather than those with diabetes [23]. Similarly, Wei et al. reported a significant association between SHR and in-hospital mortality risk in STEMI patients without diabetes [24]. The underlying mechanisms remain elusive and may be attributed to several factors. Firstly, our study focused on mortality at 90-day and 180-day post-admission, representing short-term mortality rates. Secondly, patients with diabetes, due to their adaptation to chronic inflammation and oxidative stress over time, may exhibit insensitivity to SHR [25]. Lastly, patient heterogeneity across different regions and the potential beneficial outcomes in patients with diabetes receiving insulin or other anti-inflammatory drugs should be considered [26].

During stress, the hypothalamic–pituitary–adrenal axis and the sympathetic-adrenal system are activated, increasing pro-inflammatory cytokine release, which induces stress hyperglycemia together [27]. Elevated blood glucose during an AMI is considered a primary determinant for the instability and rupture of atherosclerotic plaques, subsequently influencing an increase in coronary thrombus burden [28, 29]. Moderate stress hyperglycemia is a protective response to stress [30]. In animal shock models, the external application of hypertonic glucose can enhance cardiac output and improve survival rates [31]. However, mounting evidence indicates that stress hyperglycemia is associated with larger myocardial infarction in patients with non-obstructive coronary artery myocardial infarction and poor short- and long-term outcomes [32]. This relationship might be linked to decreased endothelium-dependent vasodilation, impaired platelet anti-aggregation, and over-activation of the sympathetic nervous system with pro-inflammatory pathways. Hyperglycemia may manifest as overactivity of the sympathetic nervous system, accompanied by glucose-mediated pro-inflammatory pathways, affecting outcomes [33, 34]. Based on the stratified analysis, we observed a increased risk of mortality in patients with a first diagnosis of AMI with an elevated SHR, especially in patients aged below 60, females, those with a BMI > 25 kg/m^2^, those with hypertension or pre-DM, without heart failure,. Thus, we should consider SHR levels clinically for patients diagnosed with AMI, especially in populations under 60, female, overweight, with hypertension or pre-DM, and without heart failure.

There are several limitations in our study. Firstly, the study design was retrospective, prohibiting definitive conclusions about causality. Secondly, being a single-center study with limited sample size, even with multivariable adjustment and subgroup analyses, potential biases due to residual confounders may persist. Third, given the inherent limitations of the MIMIC-IV database, considerations such as disease severity, baseline characteristics at admission, and sociodemographic factors like socioeconomic status and education level were not taken into account, possibly introducing potential biases in study outcomes. Additionally, selection bias may have been introduced due to the exclusion of individuals with deficiencies in glucose or HbA1c data. Finally, distinctions between specific types of acute myocardial infarctions the patients presented with and their relationships with evaluated endpoints were not made.

## Conclusions

In summary, this study revealed a J-shaped relationship between SHR and short-term outcomes in patients with a first diagnosis of AMI, underscoring the importance of optimal blood glucose control in this context. Our findings emphasize that elevated SHR levels are associated with an increased mortality rate. The inflection point for adverse prognosis with SHR is 0.9. Moreover, the causal relationship between SHR levels and short-term mortality in adult AMI patients requires further investigation.

### Supplementary Information

Additional file 1.

## Data Availability

The datasets were accessible from the MIMIC-IV (version 2.2) database. Corresponding author will provide the datasets upon reasonable request.

## Abbreviations

SHR: Stress hyperglycemia ratio
AMI: Acute myocardial infarction
HR: Hazard ratio
pre-DM: Pre-diabetes mellitus
CVD: Cardiovascular diseases
ACS: Acute coronary syndrome
PCI: Percutaneous coronary intervention
ABG: Admission blood glucose
MACCE: Major adverse cardiac and cerebrovascular events
STEMI: ST-segment elevation myocardial infarction
ICD: International classification of diseases
SQL: Structured query language
SIRS: Systemic inflammatory response
SOFA: Sequential organ failure assessment
RBC: Red blood cell
WBC: White blood cell
HbA1C: Hemoglobin A1C
BMI: Body mass index
NGR: Normoglycemia
SD: Standard deviation
CIs: Confidence intervals
VIF: Variance inflation factor
