# Supplementary material for: J-shaped relationship between stress hyperglycemia ratio and 90-day and 180-day mortality in patients with a first diagnosis of acute myocardial infarction: analysis of the MIMIC-IV database
Source: Diabetol Metab Syndr. 2024 Jun 16;16:132. doi: 10.1186/s13098-024-01380-2 (PMC11181615; doi:10.1186/s13098-024-01380-2)

**Supplementary Materials**

**Supplementary Tables**

**Table S1.** Variables query code in MIMIC-IV.

**Table S2.** Distributions of variables with missing data comparing observed complete case data to results from pooling the datasets with imputed variables from multiple imputation. Abbreviations: BMI: body mass index; WBC: white blood cell; RBC: red blood cell; PLT: platelet.

**Table S3.** Multivariable Cox regression analyses for 90-day and 180-day mortality in patients with acute myocardial infarction using raw data. Model: we adjusted for age, sex (male, female), BMI, WBC, RBC, PLT, creatinine, heart failure (yes, no), atrial fibrillation (yes, no), diabetes (yes, no), antihypertensive drugs (yes, no),antilipidemic drugs (yes, no), antiplatelets (yes, no), vasoactive (yes, no), insulin (yes, no). (n=1358) Abbreviations: BMI: body mass index; WBC: white blood cell; RBC: red blood cell; PLT: platelet.

**Table S4.** Multivariable Cox regression analyses for 90-day and 180-day mortality in patients with acute myocardial infarction. We additionally adjusted for SIRS (systemic inflammatory response) and SOFA (sequential organ failure assessment). Model: we adjusted for age, sex (male, female), BMI, WBC, RBC, PLT, creatinine, heart failure (yes, no), atrial fibrillation (yes, no), diabetes (yes, no), antihypertensive drugs (yes, no), antilipidemic drugs (yes, no), antiplatelets (yes, no), vasoactive (yes, no), SIRS and SOFA. (n=986) Abbreviations: BMI: body mass index; WBC: white blood cell; RBC: red blood cell; PLT: platelet.

**Table S5.** Multivariable Cox regression analyses for 90-day mortality in patients with acute myocardial infarction and different diabetes mellitus. Abbreviations: BMI: body mass index; WBC: white blood cell; RBC: red blood cell; PLT: platelet. Model 1: no covariates were adjusted. Model 2: we only adjusted for age, sex (male, female), BMI, cardiogenic shock (yes, no), cardiac arrest (yes, no), hypertension (yes, no). Model 3: we additionally adjusted for WBC, RBC, PLT, creatinine, heart failure (yes, no), atrial fibrillation (yes, no), antihypertensive drugs (yes, no), antilipidemic drugs (yes, no), antiplatelets (yes, no), vasoactive (yes, no), insulin (yes, no).

**Table S6.** Multivariable Cox regression analyses for 180-day mortality in patients with acute myocardial infarction and different diabetes mellitus. Abbreviations: BMI: body mass index; WBC: white blood cell; RBC: red blood cell; PLT: platelet. Model 1: no covariates were adjusted. Model 2: we only adjusted for age, sex (male, female), BMI, cardiogenic shock (yes, no), cardiac arrest (yes, no), hypertension (yes, no). Model 3: we additionally adjusted for WBC, RBC, PLT, creatinine, heart failure (yes, no), atrial fibrillation (yes, no), cardiogenic shock (yes, no), cardiac arrest (yes, no), hypertension (yes, no)，antihypertensive drugs (yes, no), antilipidemic drugs (yes, no), antiplatelets (yes, no), vasoactive (yes, no), insulin (yes, no).

Table S1. Variables query code in MIMIC-IV

| Variables | Query code |
| --- | --- |
| **Acute myocardial infarction** | 41001, 41002, 41011, 41012, 41021, 41022, 41031, 41041, 41042, 41051, 41081, 41082, 41091, 41092, I2101, I2102, I2109, I2111, I2119, I2121, I2129, I213, I214, I219, I21A1, I21A9, I222 |
| **Hypertension** | 4010, 4011, 4019, 40591, I10 |
| **Cardiogenic shock** | 78551, R570 |
| **Atrial fibrillation** | 42731; I480; I481; I4811; I4819; I482; I4820; I4821; I4891 |
| **Chronic kidney disease stage 5** | 40311; 40391; 40413; 40493; I120; I1311; I132 |
| **Cardiac arrest** | 4275, I46 |
| **Heart failure** |  |
| acute heart failure | 42821, 42823, 42831, 42833, 42841, 42843, I5021, I5023, I5031, I5033, I5043, |
| chronic heart failure | I5043,42822;42832;42842;42843;I5022;I5032;I5042;  I50812;I50813;I5084 |
| **Diabetes** | E10, E11, E13 |
| **Malignant tumor** | C220; D0462; 2330; 17331; Z85821; D0511; C44310 |
| **Vasoactive drugs** |  |
| norepinephrine | 221906 |
| dopamine | 221662 |
| epinephrine | 221289 |
| deoxyadrenaline | 221749; 229630; 229632 |
| dobutamine | 221653 |
| **Antihypertensive drugs** |  |
| angiotensin converting enzyme inhibitors | BENA10; BENA20; BENA5; CAPT1; CAPT125; CAPT25; EMLA2.5; ENAL/P2.5; ENAL/P5; ENAL1.25I; ENAL10; ENAL25I; ENAL25IVPB; ENAL5; FOSI10; FOSI20; FOSI40; LISI10; LISI2.5; LISI20; LISI5; LOTR5/20; MOEX15; MOEX7.5; QUIN20; QUIN5; RAMI/PLACIND; RAMI125; RAMI5; TRAN1; TRAN2 |
| angiotensin II receptor blockers | CAND16; CAND4; COZA25; COZAA50; ENTR24/26; ENTR49/51; ENTR97/103; IRBE150; IRBE300; LCZ696/50; LCZ696/P50; OLME20; TELM20; VALS160; VALS40; VALS80 |
| calcium-channel blockers | ADAL30; ADAL60; ADAL90; AMLO25; AMLO5; CLEV25I; EG-1962/P; FELO2.5; ISRA25; LOTR5/20; NICA2.5I; NICA20; NICA30; NICA40PM; NIFE10; NIMO30; NIMO30/P NIMO30/PS; NIMO30L; SYRINGEPD |
| clonidine | CATA1; CATA2; CLON1I; CLON5I |
| hydralazine | HYDZ10; HYDZ20I; HYDZ25; HYDZ50 |
| diuretic | FURO100DES; FURO100I; FURO100PB; FURO100S; FURO10L; FURO10S; FURO1MCGS; FURO1S; FURO20; FURO20I; FURO40; FURO40/20I; FURO40HIND; FURO40I; FURO40ILF; FURO40L FURO80,SPIR100; SPIR12.5HT; SPIR25,BISO5; CHL500I; CHL500PB; CHLO250; DYAZ1; HCTZ12.5; HCTZ25; HCTZ50; MAXZ25 |
| beta blockers | BISO5; METO12.5HT; METO1L; METO25; METO37.5; METO50; METO5I; METOSUSP; METOSUSP10L; TOPR100; TOPR25; TOPR50 |
| **Antilipidemic drugs** | ATOR/40/P; ATOR/PLACIND; ATOR10; ATOR20; ATOR40; ATOR80; ATORSTUDY; FLUV20; IMIP500I; LIPI40BN; LOVA20; MICO2P; MYC30O; MYCO2C; NYST100P; NYST100V; NYST500T; NYST5L; NYST60L; NYST60LSM; NYSTC; NYSTO; PENT10I; PENT10SYR; PENTO10I; PRAV10; PRAV10L; PRAV20; PRAV40; ROSU20; ROSU20INV; ROSU5; SIMV10; SIMV40; SIMVIND,FENO145; FENO48 |
| **Antiplatelets** | AGGRCAP; ASA300R; ASA325; ASA600R; ASA81; ASA81EC; ASAB325; ASAEC325; ASPI20.25QT; ASPI325INDP; ASPI40.5HT; ASPI81/PLB; ASPI81INDP; ASPIDESEN; FIOR,CLO025L; CLOP.25L; CLOP0.5L; CLOP01L; CLOP05L; CLOP15L; CLOP1L; CLOP2.5L; CLOP300; CLOP5L; CLOP7.5L; CLOP75; CLOP75/P; CLOP75/PIND; CLOPIND; NACLFLUSH |
| **Insulin** | 1740，27413，44340，47172，47780 |
| **Weight (Kg)** | 226512 |
| **RBC (K/µL)** | 51279 |
| **Platelet (K/µL)** | 51265 |
| **WBC (K/µL)** | 51755 |
| **Creatinine (mg/dL)** | 52024 |
| **Glucose (mg/dL)** | 220621 |
| **HbA1c (%)** | 50852 |

Table S2. Distributions of variables with missing data comparing observed complete case data to results from pooling the datasets with imputed variables from multiple imputation. Abbreviations: BMI: body mass index; WBC: white blood cell; RBC: red blood cell; PLT: platelet.

|  | Level/Unit | Number (%) with missing data | Complete case | Multiple imputation | P-value |
| --- | --- | --- | --- | --- | --- |
| BMI kg/m2 | Mean  (SD) | 29.63 | 29.69 (6.62) | 29.20 (7.56) | 0.209 |
| WBC (1000 cells/uL) | Mean  (SD) | 1.02 | 10.78 (4.66) | 10.85 (4.94) | 0.924 |
| RBC (1000 cells/uL) | Mean  (SD) | 0.31 | 4.31 (0.73) | 4.30 (0.74) | 0.877 |
| PLT (1000 cells/uL) | Mean  (SD) | 2.86 | 235.16 (78.10) | 234.38 (78.29) | 0.738 |
| Creatinine (mg/dL) | Median (interquartile range) | 0.92 | 1.00 (0.80-1.20) | 1.00 (0.80-1.20) | 0.914 |

Table S3. Multivariable Cox regression analyses for 90-day and 180-day mortality in patients with acute myocardial infarction using raw data. Model: we adjusted for age, sex (male, female), BMI, WBC, RBC, PLT, creatinine, heart failure (yes, no), atrial fibrillation (yes, no), diabetes (yes, no), antihypertensive drugs (yes, no), antilipidemic drugs (yes, no), antiplatelets (yes, no), vasoactive (yes, no), insulin (yes, no). (n=1358) Abbreviations: BMI: body mass index; WBC: white blood cell; RBC: red blood cell; PLT: platelet.

|  |  | SHR index | | |  |
| --- | --- | --- | --- | --- | --- |
|  | SHR index (continuous) | T1 | T2 | T3 | P for trend |
| **90-day mortality** |  |  |  |  |  |
| Model HR,95%CI, P | 1.46 (1.00, 2.16) 0.053 | 1 | 0.97 (0.57, 1.63) 0.901 | 1.77 (1.17, 2.67) 0.007 | 0.003 |
| **180-day mortality** |  |  |  |  |  |
| Model HR,95%CI, P | 1.44 (1.00, 2.09) 0.051 | 1 | 1.07 (0.64, 1.80) 0.795 | 1.82 (1.18, 2.82) 0.007 | 0.004 |

Table S4. Multivariable Cox regression analyses for 90-day and 180-day mortality in patients with acute myocardial infarction. We additionally adjusted for SIRS (systemic inflammatory response) and SOFA (sequential organ failure assessment). Model: we adjusted for age, sex (male, female), BMI, WBC, RBC, PLT, creatinine, heart failure (yes, no), atrial fibrillation (yes, no), diabetes (yes, no), antihypertensive drugs (yes, no), antilipidemic drugs (yes, no), antiplatelets (yes, no), vasoactive (yes, no), SIRS and SOFA. (n=986) Abbreviations: BMI: body mass index; WBC: white blood cell; RBC: red blood cell; PLT: platelet.

|  |  | SHR index | | |  |
| --- | --- | --- | --- | --- | --- |
|  | SHR index (continuous) | T1 | T2 | T3 | P for trend |
| **90-day mortality** |  |  |  |  |  |
| Model HR,95%CI, P | 1.54 (1.11, 2.13) 0.010 | 1 | 0.97 (0.57, 1.63) 0.901 | 1.77 (1.17, 2.67) 0.007 | 0.003 |
| **180-day mortality** |  |  |  |  |  |
| Model HR,95%CI, P | 1.50 (1.09, 2.06) 0.012 | 1 | 1.10 (0.68, 1.76) 0.703 | 1.78 (1.21, 2.61) 0.003 | 0.002 |

Table S5. Multivariable Cox regression analyses for 90-day mortality in patients with acute myocardial infarction and different diabetes mellitus. Abbreviations: BMI: body mass index; WBC: white blood cell; RBC: red blood cell; PLT: platelet.

Model 1: no covariates were adjusted.

Model 2: we only adjusted for age, sex (male, female), BMI, cardiogenic shock (yes, no), cardiac arrest (yes, no), hypertension (yes, no).

Model 3: we additionally adjusted for WBC, RBC, PLT, creatinine, heart failure (yes, no), atrial fibrillation (yes, no), antihypertensive drugs (yes, no), antilipidemic drugs (yes, no), antiplatelets (yes, no), vasoactive (yes, no) and insulin (yes, no).

| Diabetes status | Model 1  HR,95%CI, P | Model 2  HR,95%CI, P | Model 3  HR,95%CI, P |
| --- | --- | --- | --- |
| NGR |  |  |  |
| SHR index | 3.94 (2.60, 5.96) <0.001 | 3.91 (2.27, 6.73) <0.001 | 2.47 (1.35, 4.53) 0.003 |
| T1 | Ref | Ref | Ref |
| T2 | 0.99 (0.40, 2.48) 0.983 | 1.05 (0.42, 2.64) 0.912 | 0.98 (0.38, 2.50) 0.965 |
| T3 | 3.40 (1.52, 7.63) 0.003 | 2.51 (1.10, 5.74) 0.029 | 2.04 (0.97, 4.79) 0.056 |
| P for trend | <0.001 | <0.001 | 0.034 |
| Pre-DM |  |  |  |
| SHR index | 12.67 (5.69, 28.19) <0.001 | 10.64 (4.33, 26.11) <0.001 | 8.83 (3.06, 25.47) <0.001 |
| T1 | Ref | Ref | Ref |
| T2 | 2.85 (0.90, 9.10) 0.076 | 3.09 (0.95, 10.06) 0.061 | 2.67 (0.77, 9.28) 0.121 |
| T3 | 8.51 (2.86, 25.29) <0.001 | 7.73 (2.45, 24.35) <0.001 | 6.90 (1.98, 24.02) 0.002 |
| P for trend | <0.001 | <0.001 | 0.002 |
| DM |  |  |  |
| SHR index | 2.03 (1.37, 3.00) <0.001 | 1.74 (1.14, 2.64) 0.010 | 1.94 (1.42, 2.66) <0.001 |
| T1 | Ref | Ref | Ref |
| T2 | 0.39 (0.18, 0.85) 0.018 | 0.42 (0.19, 0.92) 0.031 | 0.82 (0.51, 1.32) 0.422 |
| T3 | 1.64 (1.05, 2.56) 0.031 | 1.42 (0.90, 2.23) 0.129 | 1.98 (1.35, 2.90) <0.001 |
| P for trend | 0.025 | 0.099 | <0.001 |

Table S6. Multivariable Cox regression analyses for 180-day mortality in patients with acute myocardial infarction and different diabetes mellitus. Abbreviations: BMI: body mass index; WBC: white blood cell; RBC: red blood cell; PLT: platelet.

Model 1: no covariates were adjusted.

Model 2: we only adjusted for age, sex (male, female), BMI, cardiogenic shock (yes, no), cardiac arrest (yes, no), hypertension (yes, no).

Model 3: we additionally adjusted for WBC, RBC, PLT, creatinine, heart failure (yes, no), atrial fibrillation (yes, no), cardiogenic shock (yes, no), cardiac arrest (yes, no), hypertension (yes, no)，antihypertensive drugs (yes, no), antilipidemic drugs (yes, no), antiplatelets (yes, no), vasoactive (yes, no).

| Diabetes status | Model 1  HR,95%CI, P | Model 2  HR,95%CI, P | Model 3  HR,95%CI, P |
| --- | --- | --- | --- |
| NGR |  |  |  |
| SHR index | 3.38 (2.23, 5.14) <0.001 | 3.26 (1.90, 5.58) <0.001 | 2.30 (1.27, 4.19) 0.006 |
| T1 | Ref | Ref | Ref |
| T2 | 0.91 (0.41, 1.98) 0.804 | 0.99 (0.45, 2.16) 0.971 | 0.97 (0.43, 2.15) 0.931 |
| T3 | 2.79 (1.40, 5.55) 0.004 | 2.16 (1.07, 4.37) 0.032 | 1.86 (0.89, 3.88) 0.0981 |
| P for trend | <0.001 | 0.008 | 0.038 |
| Pre-DM |  |  |  |
| SHR index | 10.47 (4.92, 22.24) <0.001 | 9.02 (3.88, 20.99) <0.001 | 8.11 (3.08, 21.35) <0.001 |
| T1 | Ref | Ref | Ref |
| T2 | 2.89 (1.12, 7.45) 0.028 | 3.41 (1.30, 8.95) 0.013 | 2.65 (0.96, 7.31) 0.060 |
| T3 | 6.49 (2.59, 16.26) <0.001 | 6.34 (2.42, 16.62) <0.001 | 5.30 (1.96, 14.27) 0.001 |
| P for trend | <0.001 | <0.001 | 0.001 |
| DM |  |  |  |
| SHR index | 2.69 (2.07, 3.48) <0.001 | 2.32 (1.75, 3.09) <0.001 | 1.87 (1.39, 2.52) <0.001 |
| T1 | Ref | Ref | Ref |
| T2 | 0.44 (0.22, 0.88) 0.020 | 0.45 (0.22, 0.92) 0.028 | 0.90 (0.59, 1.36) 0.611 |
| T3 | 1.69 (1.11, 2.57) 0.014 | 1.47 (0.96, 2.26) 0.074 | 1.97 (1.39, 2.80) <0.001 |
| P for trend | 0.011 | 0.053 | <0.001 |

Figure S1. Kaplan-Meier survival analysis curves for all-cause mortality. Footnote SHR index (two groups according to the inflection point). Kaplan-Meier curves showing cumulative probability of all-cause mortality according to groups at 90 days (A), and 180days (B). The adjustment strategy is the same as the Model 3.


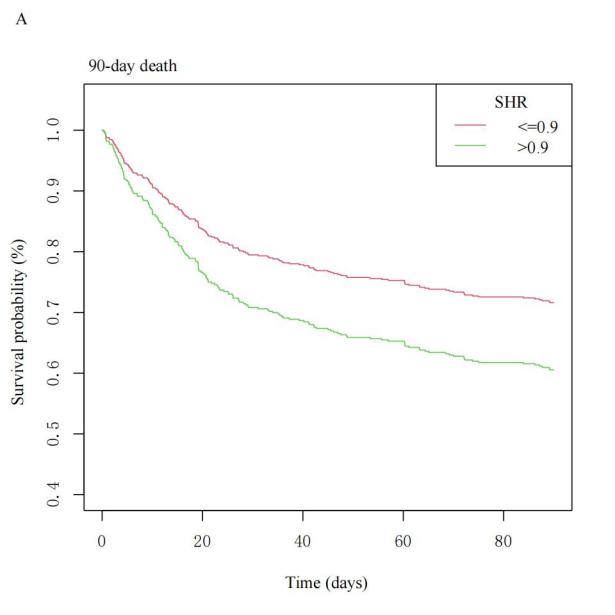

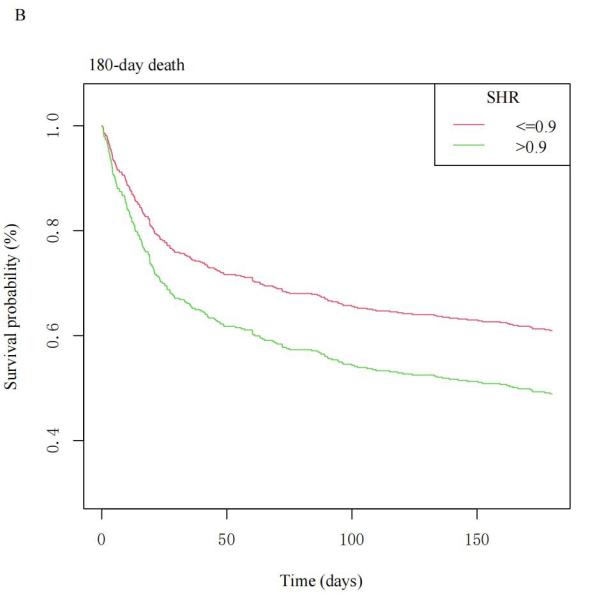

Supplement: Supplementary file 1 — Additional file 1. [file 13098_2024_1380_MOESM1_ESM.docx]
